# Retained Placenta

**Published:** 1876-02

**Authors:** 


RETAINED PLACENTA.—In reference to some cases of retained placenta that had been treated by forcible removal, which he regards as a dangerous practice, Dr. Lineard, of Caen, calls attention to the fact that many years ago he published a simple procedure, which he has always found as effectual as it is safe and easy, and which is also a very efficacious means for the prevention of after-pains and uterine hemorrhage. It consists in the injection of the umbilical vein with cold water. A clean section should first be made, so as to shorten the cord, which should not be more than from twenty to thirty centimetres in length. A syringe, containing at least 150 grammes, and having long fixed canula, should be employed. The colder the water used, the less is the quantity that need be injected, so that while 150 grammes suffice at the ordinary temperature of winter, twice or thrice as much may be required in summer.—*Gaz. des Hop.*

